# Pattern Mixture Sensitivity Analyses via Multiple Imputations for Non‐Ignorable Dropout in Joint Modeling of Cognition and Risk of Dementia

**DOI:** 10.1002/sim.70040

**Published:** 2025-03-13

**Authors:** Tetiana Gorbach, James R. Carpenter, Chris Frost, Maria Josefsson, Jennifer Nicholas, Lars Nyberg

**Affiliations:** ^1^ Department of Statistics, Umeå School of Business, Economics and Statistics Umeå University Umeå Sweden; ^2^ London School of Hygiene and Tropical Medicine London UK; ^3^ MRC Clinical Trials Unit at University College London London UK; ^4^ Department of Integrative Medical Biology Umeå University Umeå Sweden; ^5^ Umeå Center for Functional Brain Imaging Umeå University Umeå Sweden; ^6^ Department of Radiation Sciences Umeå University Umeå Sweden

**Keywords:** linear mixed effect model, multiple imputation, pattern mixture model, proportional hazards model, sensitivity analysis

## Abstract

Motivated by the Swedish Betula study, we consider the joint modeling of longitudinal memory assessments and the hazard of dementia. In the Betula data, the time‐to‐dementia onset or its absence is available for all participants, while some memory measurements are missing. In longitudinal studies of aging, one cannot rule out the possibility of dropout due to health issues resulting in missing not at random longitudinal measurements. We, therefore, propose a pattern‐mixture sensitivity analysis for missing not‐at‐random data in the joint modeling framework. The sensitivity analysis is implemented via multiple imputation as follows: (i) multiply impute missing not at random longitudinal measurements under a set of plausible pattern‐mixture imputation models that allow for acceleration of memory decline after dropout, (ii) fit the joint model to each imputed longitudinal memory and time‐to‐dementia dataset, and (iii) combine the results of step (ii). Our work illustrates that sensitivity analyses via multiple imputations are an accessible, pragmatic method to evaluate the consequences of missing not at‐random data on inference and prediction. This flexible approach can accommodate a range of models for the longitudinal and event‐time processes. In particular, the pattern‐mixture modeling approach provides an accessible way to frame plausible missing not at random assumptions for different missing data patterns. Applying our approach to the Betula study shows that worse memory levels and steeper memory decline were associated with a higher risk of dementia for all considered scenarios.

**Abbreviations**
MAR: missing at random
MNAR: missing not at random

## 1 Introduction

Joint modeling (JM) of longitudinal and time‐to‐event data [1, 2, 3, 4] has become pivotal in various clinical applications, such as personalized prediction of time to a clinical milestone [5, 6, 7, 8]. In the cognitive aging literature, joint modeling has proven particularly useful in evaluating the association between various longitudinal health trajectories and subsequent dementia risk. For example, joint modeling has been used to study the association between dementia risk and cognition [9, 10, 11] and brain imaging measures [12].

The present study is motivated by the Swedish Betula study, [13, 14] which aims to investigate how memory functions change in healthy and pathological aging, to identify risk factors for dementia, and to determine early preclinical signs of dementia. Betula is a prospective cohort study that collected health data from over 4 000 participants, with some individuals' health being followed for up to 25 years. One challenge in Betula, as well as in many (if not all) longitudinal aging studies, is missing longitudinal data due to participant attrition. Note here that in the Betula study, dementia diagnoses were obtained from medical records even after dropout, so the time‐to‐event outcome is assumed to be available for everyone. Missing longitudinal data due to dropout is particularly pronounced among older cohorts, where an individual's current declining health may influence their decision to withdraw from the study. As a result, study withdrawal might be related to missing longitudinal data that was initially intended to be used in the analysis even given all available data. In such a case, missing longitudinal health‐related data are called missing not at random (MNAR) [15]. The cognitive aging literature has long acknowledged that people who drop out of studies typically exhibit (prior to study dropout) worse cognitive performance and steeper decline compared to those who remain, [16, 17, 18, 19] and has seen this as an indication of MNAR data. Properties of statistical analyses of such data rely on the assumptions made about the missing data mechanism, that is, the relation of the fact that data is missing to available and not available for the analyses data (Chapter 1.3 in Little and Rubin [20]). Conventional approaches assume missing at random (MAR) missing data mechanism, which means that the fact that data is missing is not related to the missing values given the available data [15]. For example, memory is missing at random if its missingness does not depend on missing memory measurements given other available measurements that can be incorporated into the analyses. Since the conventional methods disregard the MNAR nature of the missing data, they may yield biased inferences.

For example, consider the task of predicting dementia risk for a new subject with regularly monitored memory measures based on a model built from prospective data with possibly MNAR memory data. Only considering the observed data under the assumption of MAR data in the model‐building step may lead to falsely overestimating memory performance in the general population [21, 22]. This bias may distort inferences on the relationship between memory and dementia risk, consequently resulting in misleading predictions of dementia risk for new patients. To understand the extent to which this might be an issue, one has to explore the robustness of inferences to assumptions about missing data mechanism. Previous work on MNAR longitudinal data within the JM framework for longitudinal and survival data has either considered a shared parameter modeling approach, in which the hazard of dropout is modeled as dependent on the last observed value and on the random effects, [23] or employed a competing risk analysis, where dropout is modeled as a competing event to dementia [24]. However, the former relies on untestable assumptions about the latent random effect distribution, while the latter does not directly account for the possibility of progression to dementia after dropout. A fully Bayesian approach to analyses with missing data is described in Linero and Daniels [25]. This approach has been applied in the joint modeling framework but with an application for longitudinal normal and binary, but not survival, data in Gaskins et al. [26] However, we are not aware of any applications of a fully Bayesian approach to joint modeling of longitudinal and survival data.

Unfortunately, in longitudinal aging studies, the researchers can not control all mechanisms that lead to data being missing. Additionally, assumptions about the exact nature of the missingness mechanism are inherently untestable from the observed data only. In these settings, sensitivity analyses can aid in understanding the robustness of inferences and predictions for a range of contextually plausible assumptions about the (possibly MNAR) data [27, 28]. Herein lies a dual challenge for statisticians: Framing missing data assumptions comprehensibly for our collaborators, and incorporating such assumptions into the analytical framework. Previously, Carpenter and colleagues [29] argued that pattern‐mixture imputation models provide an accessible approach for sensitivity analyses and that multiple imputation is a practical approach to analyzing incomplete data while incorporating various assumptions about missing data.

In this article, we extend the pattern‐mixture multiple imputation approach to sensitivity analyses for missing not‐at‐random data to joint models. We apply the approach to explore the relationship between longitudinal trends in episodic memory and dementia risk using the Betula data under various assumptions about the missing data. First, we perform multiple imputations of missing memory scores using the information about the dropout time via a pattern‐mixture approach. We consider various assumptions about missing data, reflecting different scenarios about post‐dropout memory decline acceleration. Secondly, we fit the joint model to each completed data set containing the observed and the imputed data and use standard multiple imputation techniques for inference. Finally, we compare inferences from the joint model under different assumptions about post‐dropout memory decline acceleration.

The paper is structured as follows: Section 2 briefly introduces the Betula study, Section 3 sets out our proposed methodology, which we apply in our analysis of the Betula data set in Section 4. We conclude with a discussion of the practical and methodological findings and implications for future work in Section 5.

## 2 The Betula Study

The Betula study is a population‐based prospective cohort study of aging, memory, and dementia conducted at Umeå University, Sweden [13, 14]. The Betula study was approved by the Regional Ethical Vetting Board at Umeå University and written consent was obtained from all participants. Participants were randomly sampled from the population registry and were between 25 and 95 years old at enrollment. Key inclusion criteria were being dementia‐free at enrollment, having Swedish as a mother tongue, and having no vision/hearing impairment; full details are described in detail in Nilsson et al. [13]. To date, six major waves of data collection, T1–T6, have been conducted, with approximately five years between successive waves. At each wave, participants underwent an extensive health examination, completed a questionnaire about socioeconomic factors, and underwent cognitive assessments. As Table 1 shows, the Betula sample consists of six cohorts, S1–S6, that started participation in Betula at different waves and were scheduled for varying numbers of measurement waves.

**TABLE 1:** Number of participants of the Betula study with available episodic memory score before dementia diagnosis, by Betula cohort (S1–S6) and follow‐up waves (T1–T6)^a^.

Betula wave →	T1	T2	T3	T4	T5	T6
Betula cohort ↓	1988–1990	1993–1995	1998–2000	2003–2005	2008–2010	2013–2014
S1	999	824	620	486	355	218
S2		975	585		7	
S3		950	715	552	383	229
S4			547		1	
S5				557		
S6					356	63

*Note:* Empty cells correspond to no scheduled observations.

^a^ The number of participants in Table 1 is comparable to the number of memory examinations in Table 1 in Nyberg et al. [14] The difference is due to some participants not having a score for all five tests used to calculate the episodic memory score. Also, the memory scores for 7 participants of Betula Cohort 2 at Wave 5 and one participant of Betula Cohort 4 at Wave 5 were available but not scheduled by the initial Betula design. We include these 8 data points in our analyses. Additionally, 10 participants started participation in Betula later than their scheduled first measurement waves. Thus, the sum of the number of participants in the first scheduled wave for each Betula cohort is 4 384, not 4 394.

In this article, we consider episodic memory as the longitudinal outcome. Episodic memory was assessed by calculating the sum of scores for five episodic memory tasks: Immediate free recall of 16 short sentences without enactment, immediate free recall of 16 short sentences with enactment, delayed cued recall of nouns from the previously presented sentences without enactment, delayed cued recall of nouns from the enacted sentences, and immediate free recall of a list of 12 orally presented nouns. The memory scores range from 0 to 76, with lower scores indicating worse memory [13]. The number of people with available episodic memory scores is presented in Table 1. If the score of one or more of the five component tests is missing for a participant at a follow‐up episodic memory is defined as missing at that follow‐up wave. There were 198 such data points: 87 had missing scores for four tests; 7 for three tests; 16 for two tests, and 88 for one test.

Nyberg et al. [14] thoroughly described dementia assessment in Betula, which we briefly summarize here. The diagnostic procedure was based on medical records and a health and memory assessment. To identify dementia onset during Betula data collection, participants that (a) had a mini‐mental state examination score below 24 or a decline of at least 3 points from the score of the previous Betula visit or (b) had their cognitive performance declined from high to normal/low or from normal to low from the previous testing occasion or (c) reported memory dysfunction or (d) had a cognitive or behavioral variation that implies neurocognitive impairment were identified and were referred for an extended dementia evaluation. This evaluation was based on the DSM‐IV classification core criteria for dementia [30]. The year when the core criteria for dementia were met was defined as dementia onset. After a participant had withdrawn from the Betula study, information about their dementia diagnosis and death was available from medical records. The dementia evaluations were updated roughly every five years, and this paper incorporates the latest updates available up to the year 2 022. For around 60% of dementia cases, dementia onset started after they had stopped participating in the Betula study.

Of 4 394 participants in the current study, 2 179 had some scheduled observations missing because they withdrew or died (679 out of 4 394 participants died less than five years after their last available memory measurement). The most frequent reasons for withdrawal were health issues, unwillingness to participate, and relocation from the catchment area. Some intermittent measurements were missing for 59 out of 4 394 participants before their last follow‐up (see Table B1 for the presentation of the patterns of observed data and missing intermittent measurements).

## 3 Methods

We propose a sensitivity analysis approach based on multiple imputation [31] in the joint modeling framework. Multiple imputation consists of replacing each missing value several times according to some assumed distribution of missing values given observed data, estimating the model of interest to each completed data set, and pooling the estimates from the completed data sets. There are various ways to perform imputations as long as imputations are proper, [31] for example, in case of multivariate missing using a joint model of missing and observed data or a fully conditional specification [32]. When data are MNAR, the observed data alone can be consistent with numerous joint distributions of the observed and unobserved data (full‐data distributions) [33]. Therefore, a natural sensitivity analysis approach begins with analyses of the observed data, assuming MAR. Analyses are performed under the primary model of interest, commonly referred to as a substantive model within the multiple imputation framework [29]. Then, one explores how inferences on the substantive model change when data are imputed according to the various departures from MAR. For example, in cognitive aging studies a common assumption is that individuals dropping out have worse memory than corresponding members of their study cohort who do not drop out. Therefore, it is natural to investigate the robustness of inferences from the observed data to additional cognitive decline after dropout, beyond that which would be predicted from individuals who do not drop out. We, therefore, propose a sensitivity analysis within the joint modeling framework, adhering to the following conventional multiple imputation steps [1, 5, 31, Chapter 1.5]:

- Fit the substantive joint model to the observed data assuming MAR data.
- Specify the range of plausible full‐data distributions under MNAR data parameterized by a sensitivity parameter Δ.
- For each considered full‐data distribution (each value of Δ) perform multiple imputation as follows:
  1. Impute each missing value K times according to some imputation model to obtain K completed data sets containing both the observed and the imputed data.
  2. Estimate the parameters of interest by fitting the substantive joint model to each of K completed data sets.
  3. Combine the K parameter estimates into one pooled estimate [31, 34].
- Compare inferences and predictions on the substantive joint model across the range of sensitivity parameter Δ values.

The details of the substantive model are presented in Section 3.1 and the pattern‐mixture‐based imputation model used in step C is presented in Section 3.2. In Appendix A, we provide the rules for combining estimates in step 3.

### 3.1 Substantive Model: A Joint Model

Within the joint modeling framework, longitudinal trajectories are typically represented by linear mixed‐effects models (LME) [35], while the time‐to‐event data are described by a proportional hazard, a competing risk, or a multi‐state model [3]. To represent the link between the longitudinal and time‐to‐event data, certain aspects of the longitudinal memory trajectory, that is, the level and/or rate of change, are included as predictors in the time‐to‐event model (for details see Chapter 4 in Rizopoulos [3] figure A).

In the present study, we specify an LME submodel for the longitudinal outcome and a proportional hazards model for the time‐to‐event outcome. Let $y_{i}(t)$ denote the observed longitudinal outcome and $h_{i}(t)$ denote the hazard of the event for participant i at time t, i=1,…,N. Time t, for example, can represent age or time from enrollment in the study. Then the joint model is:

(1) $$\begin{array}{l} \left\{\begin{array}{l} y_{i}(t)=m_{i}(t)+\varepsilon_{i}(t),\\ m_{i}(t)=x_{i}^{⊤}(t)\beta +z_{i}^{⊤}(t)b_{i},\\ b_{i}\overset{iid}{∼}𝒩(0,D),\;\text{independent of}\;\varepsilon_{i}(t)\overset{iid}{∼}𝒩(0,\sigma^{2}),\\ h_{i}(t|{ℳ}_{i}(t),w_{i})=h_{0}(t)\exp\left(\gamma^{⊤}w_{i}+\alpha^{⊤}f\left(m_{i}\right)\right) \end{array}\right. \end{array}$$

Here, the observed outcome $y_{i}(t)$ is the sum of the true value of the longitudinal outcome at timepoint t, $m_{i}(t),$ and the measurement error, $\varepsilon_{i}(t).$ The function $m_{i}(t)$ models the mean memory score given covariates $x_{i}(t)$ and $z_{i}(t)$ and random effects $b_{i}.$ The design vectors $x_{i}(t)$ and $z_{i}(t)$ are for the fixed and the random effects, respectively, including baseline and time‐varying covariates. A vector of subject‐specific random effects $b_{i}$ is independent of the error term $\varepsilon_{i}(t).$ The error terms are mutually independent and normally distributed random variables with zero mean and variance $\sigma^{2}.$

In the time‐to‐event submodel, the hazard of the event for individual i at time t, $h_{i}(t|{ℳ}_{i}(t),w_{i})=\lim_{dt\to 0}\mathrm{Pr}(t\leq T_{i}^{*}<t+dt|T_{i}^{*}\geq t,{ℳ}_{i}(t),w_{i})/dt,$ where $T_{i}^{*}$ represents the true time of the event, depends on baseline covariates $w_{i}$ and the history of the true unobserved longitudinal process up to time t, ${ℳ}_{i}(t)=\{m_{i}(s),0\leq s<t\}$ (here s denotes time before t). The term $h_{0}(t)$ represents the baseline hazard at time t. The term $\alpha^{⊤}f(m_{i})$ quantifies the relationship between the hazard of the event at time t and the true longitudinal process. The choice of $f(m_{i})$ depends on the research question. For example, $f(m_{i})$ can be the true value of the longitudinal outcome $m_{i}(s)$ at time point s, the slope of the trajectory $m_{i}^{\prime }(s),$ a vector of the true value and slope, ${\left(m_{i}(s),m_{i}^{\prime }(s)\right)}^{T},$ the random effects $b_{i},$ etc. (Chapter 4 in Rizopoulos [3]).

Joint models can be fitted in R [36] using a frequentist approach with R‐packages such as JM, joineR, lcmm, frailtypack, rstanarm or with a Bayesian approach using JMbayes, JMbayes2 and bamlss, see Cekic et al. [4] for the comparison of packages' functionality. These software packages analyze the observed data and, therefore, provide valid conclusions when longitudinal outcomes are missing at random. However, if longitudinal outcomes are thought to be missing not at random, these approaches are generally invalid and might introduce biased estimates [20].

### 3.2 Imputation Model

The imputation steps B and C in Section 3 require specification of the range of plausible full‐data distributions and the corresponding imputation models assuming data are MNAR. Note that, we consider MNAR longitudinal outcome data (i.e., MNAR episodic memory data), and non‐informative censoring in the time‐to‐event data. The pattern‐mixture model provides an intuitive approach for framing sensitivity analyses: The model specifies that people with different patterns of missing data (usually time in the study) might have different full‐data distributions (see (12) in Little, 1995 [37] and prior application to the Betula data in Josefsson et al. [38]). In longitudinal aging studies, it may be plausible to assume an MNAR process whereby people with shorter follow‐up times have worse memory performance, both in level and rate of change, compared to those who stay in the study longer. To incorporate this in the imputation model we start with some additional notation.

Let $y_{i}={\left(y_{i1},\ldots ,y_{in_{i}}\right)}^{T}$ denote all $n_{i}$ longitudinal data scheduled to be observed for participant i at times $t_{i}={\left\{t_{i1},\ldots ,t_{in_{i}}\right\}}^{T},$ i=1,…,N. Let $r_{ij},j=1,\ldots ,n_{i},$ be an indicator of data being observed, that is, $r_{ij}=1$ if $y_{ij}$ is observed and 0 otherwise. Let $r_{i}={\left\{r_{i1},\ldots ,r_{in_{i}}\right\}}^{⊤}.$ Observed memory‐related data for participant i includes the measured covariates, the observed outcomes, and the indicators of the observed outcomes $\{x_{i}{\left(t_{i}\right)}^{⊤},z_{i}{\left(t_{i}\right)}^{⊤},\{y_{ij},j:\;r_{ij}=1\},r_{i}^{⊤}\}.$ For each participant i that drops out, we define the dropout time $t_{iF_{i}}$ as the time of the last observed measurement, $F_{i}=\max\{j:\;r_{ij}=1\}.$ Following the pattern‐mixture framework of analyses with missing data, the imputation model is the joint distribution of all data and missingness where the distribution of the longitudinal data depends on the missing data pattern $d_{i}.$ As such, $p(y_{i}(t),h_{i}(t),b_{i},d_{i}|x_{i}(t),z_{i}(t))=p(y_{i}(t)|b_{i},d_{i},x_{i}(t),z_{i}(t))p(b_{i}|d_{i},x_{i}(t),z_{i}(t))p(d_{i}|x_{i}(t),z_{i}(t)).$ Therefore, we impute missing longitudinal data using the posterior for the longitudinal outcome from the model (1) with $m_{i}(t)$ allowed to differ between the missing data patterns:

(2) $$\begin{array}{l} m_{i}(t)=x_{i}^{⊤}(t)\beta^{d_{i}}+z_{i}^{⊤}(t)b_{i}+\Delta {\left(t-t_{iF_{i}}\right)}_{+} \end{array}$$

Here, the information about the dropout time is used for imputations by allowing memory to depend on the missing data pattern. Additionally, because the missing longitudinal data are imputed using the joint model, the imputations are informed by both the longitudinal and survival data. Traditionally, the pattern‐mixture approach involves stratifying data based on $r_{i}$ [31]. However, in studies with a complex design and multiple follow‐ups, as in the Betula study, the response indicator vector, $r_{i}$, can take a large number of values, each representing a distinct observed data pattern. Thus, there may be only a few individuals (and hence observations) for some values of the vector $r_{i}.$ Consequently, some model parameters might be unidentified from the data. The solution employed here is to group missing data indicators $r_{i}$ into missing data patterns $d_{i}$ based on the number of scheduled measurements and length of stay in the Betula study before dropout (for example, 25 years for the participants that stayed in the Betula study for all 6 waves or 10 years for participants that stayed for 3 waves.)

**FIGURE D1:**
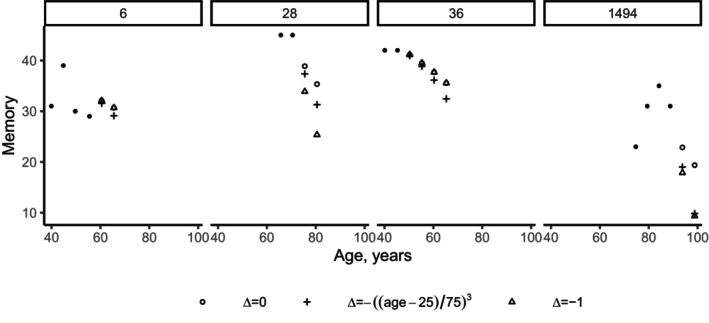
Observed (filled circles) and posterior means of missing memory scores per participant and timepoint where data are imputed for different values of Δ for some participants with missing memory measurements and at least two observed memory scores. Participants 6 and 36 dropped out before 60 years old therefore, posterior means of their imputed memory for Δ=0 and Δ=1 are the same, while a small decrease of memory is introduced when Δ is age‐varying. Since Δ is closer to −1 for older ages when considering age‐varying Δ, imputations for older ages under age‐varying Δ are closer to the imputations under Δ=−1.

To allow for MNAR outcome data, we introduce the sensitivity parameter Δ. The sensitivity parameter represents the change in the linear slope of the longitudinal outcome after the last follow‐up (where ${\left(t-t_{iF_{i}}\right)}_{+}=t-t_{iF_{i}}$ if $t>t_{iF_{i}}$ and 0 otherwise). If Δ=0, then one assumes that given the missing data pattern $d_{i},$ the trajectory of longitudinal outcome does not change after dropout, i.e., data are consistent with MAR given the missing data pattern. If Δ is negative, then one assumes that the decline of the longitudinal outcome after dropout is steeper than before the dropout. A negative Δ can also be thought of as an “additional decline” compared to Δ=0: The longitudinal outcome after dropout changes on average by Δ more per time unit compared to Δ=0. The value of Δ might also depend on time or the value of other covariates. Figure D1 shows an example of how different sensitivity parameter values affect the imputed values.

Since Δ describes how the unobserved data differs from the observed data, it is undefined from the observed data and is a sensitivity parameter (see Section 8.4.2 in Daniels and Hogan [27] and p. 246 in Carpenter et al. [29]). Imputing missing data and fitting the joint model for various values of Δ allows studying the sensitivity of inferences to different changes in linear slope after the last observed follow‐up.

## 4 Empirical Study

We now use the proposed procedure to study the association between episodic memory and the risk of dementia five years later in the Betula study [13, 14]. The code for all analyses is provided in the Supporting Information.

### 4.1 Data Preparation

The data preparation steps before the analyses are described in detail in Appendix B. Briefly, memory observations after dementia diagnosis are deleted because we focus on predicting dementia risk from the preceding memory trajectory. For participants not diagnosed with dementia, age at censoring is defined as either the age of death or the age at the latest dementia evaluation, whichever comes first (1 253 and 2 140, respectively, out of 4 394 participants). The latest dementia evaluation was performed between 2 013 and 2 021 after most Betula follow‐up visits. For 74 participants for whom information about the most current dementia and death evaluation was unavailable, age at censoring is defined as the age at the last memory assessment plus 0.003 years (approximately one day). Individuals with early dementia onset (before 65 years old, 11 participants) are excluded since they likely have a more aggressive disease progression, which is hard to capture due to the long period of 5 years between the Betula waves. We also excluded 63 participants with missing information about their education (83 memory measurements are excluded due to missing education). Such listwise deletion provides valid estimates if the probability of education being missing does not depend on memory scores (the outcome) or the hazards of dementia. As a result, the analytical sample consists of 9 339 observed memory measures for 4 331 participants and 2 246 memory measures to be imputed for 1 443 participants. Out of the 1 443 participants with missing data, 1 027 participants had only one measurement to impute.

### 4.2 The Substantive Model: Joint Model

We are interested in studying the relationship between level and change in memory and the risk of dementia five years later. The joint model then becomes (3):

(3) $$\begin{array}{l} \left\{\begin{array}{l} y_{i}(t)=m_{i}(t)+\epsilon_{i}(t)\\ m_{i}(t)=f(t)+x_{i}^{⊤}(t)\beta +b_{0i}+b_{1i}(t-\bar{t})\\ \left(b_{0i},b_{1i})∼𝒩(0,D),\;\varepsilon_{i}(t)∼𝒩(0,\sigma^{2}\right)\\ h_{i}(t|{ℳ}_{i}(t),w_{i})=h_{0}(t)\exp\left(\gamma^{⊤}w_{i}+\alpha_{1}m_{i}(t-5)+\alpha_{2}m_{i}^{\prime }(t-5)\right) \end{array}\right. \end{array}$$

Here, $y_{i}(t)$ represents the memory score for participant i at age t years, f is a smooth function of t, $\bar{t}$ is the mean age at memory assessments with observed memory scores, that is, $\bar{t}=\frac{\sum_{i=1}^{N}\sum_{j=1}^{n_{i}}r_{ij}t_{ij}}{\sum_{i=1}^{N}\sum_{j=1}^{n_{i}}r_{ij}},$ where $t_{ij}$ is the age of participant i at their jth memory assessment. The vector of covariates, $x_{i}(t)$ for participant i consists of sex, years of education, an indicator of the first measurement to adjust for practice effects (improvement of performance due to repeated exposure to a cognitive test), an interaction between the first measurement indicator and age to allow for practice effects that change with age. Birth cohort, a continuous variable defined as the difference between a participant's birth year and 1 937, is also included to control for cohort effects. The covariates $w_{i}$ consist of sex and years of education. The smooth function f is approximated by natural cubic splines with 3 degrees of freedom. Model selection for the longitudinal submodel is described in Appendix C. Note that the choice of a five‐year lag is unrelated to Betula's design but driven by the research question.

The joint model (3) is fitted using the R‐package JMbayes2 [39] with three chains (default setting), 10 000 warm‐up iterations and 100 000 iterations afterward. We use every 10th iteration after warm‐up for posterior inferences. The baseline hazard is approximated using B‐splines (the package's default). To ensure model convergence, we have checked that the scale reduction factor was close to 1, acceptance rates were not too low or too high, and that the trace plots showed the well‐mixed chains.

### 4.3 Imputation Model

We impute missing longitudinal memory measurements after dropout for people who withdrew from the study. Note that we do not impute intermittent observations. Since we are interested in the association of memory and the hazard of a subsequent event, we do not impute longitudinal measurements after the occurrence of the event. Similarly, we do not impute longitudinal measurements after death because considering memory after death is not conceptually reasonable.

**FIGURE 1:**
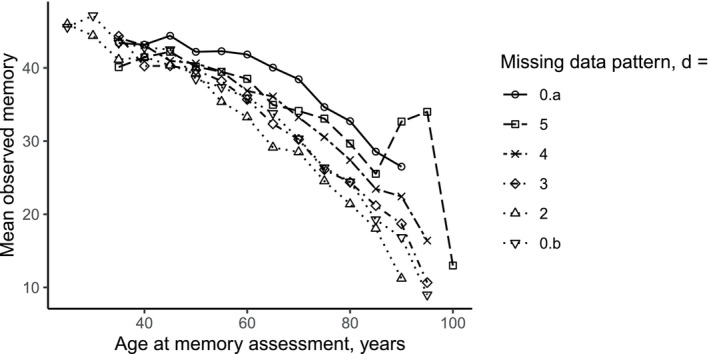
Mean observed memory scores before dementia versus age at memory assessment for each considered missing data pattern d in the Betula data set (see the text for definitions of the patterns). A point at age x represents the mean of observed memory scores among the participants aged between x−2.5 years and x+2.5 years at the time of memory assessment, who belong to a specific missing data pattern and are not yet demented at age x. For example, the mean observed memory score for individuals in pattern d=2, aged between 87.5 and 92.5 years old and not demented at this assessment is approximately 11.

To specify a pattern‐mixture full‐data distribution according to model (2) and the corresponding imputation models, one has first to define the missing data patterns. In this study, we consider six missing data patterns on the length of participants' stay in the Betula study and their respective Betula cohort:

- Patterns d=0.a and d=0.b represent people who do not withdraw before their last scheduled measurement wave. Pattern d=0.a includes Betula cohorts S1 and S3, which were scheduled for five or six measurement waves, respectively (see Tables 1, B1 and B2). Pattern d=0.b includes the remaining Betula cohorts that were scheduled for only one or two time points by study design. We separate d=0.a and d=0.b since, as Figure 1 shows, the memory profile for d=0.b is very different from the profile for participants in d=0.a, where the latter is a highly selected group who stayed in Betula for 20–25 years. As Table 1 shows, since memory measurements after dementia were not used in the analyses and intermittent missing memory values infrequently occur, the total number of memory observations is sometimes less than the scheduled number of measurement waves.
- d=j, j=3,4,5 for the participants from longitudinal Betula cohorts S1 and S3 that drop out after their jth scheduled measurement (Betula wave Tj for S1 and T(j+1) for S3).
- d=2 for dropouts from all Betula cohorts with memory measurements available from one or two Betula waves. Note that this pattern includes participants with one memory measurement to allow identification of the LME model from the observed data. The parameters of the LME model with random intercept and slope are not identified for a pattern with only one data point per subject available.

Information about participants' Betula cohort was used to define missing data patterns since cohorts were scheduled for varying numbers of measurements by the Betula design. Other groupings into missing data patterns are possible, such as those based on age at dropout or the number of available measurements. However, since Betula participants entered the study at different ages and were scheduled for varying numbers of measurements, we believe that the length of stay, approximated by the time between the first and last observations, is more representative of a dropout pattern in the Betula study (as memory patterns for the participants with only two observations by design might differ from those who were scheduled for six measurements but dropped out after two).

As noted above, 59 participants had one or two intermittent missing measurements (based on “Observed waves before dementia diagnosis” used in the analyses, see Table B1). In total, intermittent missing appeared for less than 1% of scheduled measurements.

Figure 1 shows that, on average, participants with fewer observations had worse memory than those of the same age who remained in the study longer. The notable “jump” observed in the d=5 group at older ages is due to one individual. Table 2 shows that among patterns 0.a,5,4, and 3 in the Betula data, groups with fewer memory observations available, a higher proportion of participants diagnosed with dementia, and individuals tended to be older at enrollment compared to groups with more observations.

**TABLE 2:** Descriptive characteristics for the Betula data per missing data pattern.

Missing data pattern	*N*	Male	Demented	Deceased	$\bar{\text{Education}}$	$\bar{\text{Age enrol.}}$	$\bar{\text{Age dementia}}$	$\bar{\text{Age censor.}}$
0.a	451	210 (47%)	25 (6%)	39 (9%)	12.1	48	79.7	74.1
5	142	67 (47%)	22 (15%)	47 (33%)	10.7	51	80.9	76.8
4	308	123 (40%)	60 (19%)	149 (48%)	10.0	59	81.2	79.3
3	326	147 (45%)	79 (24%)	210 (64%)	9.4	63	81.1	79.2
2	1 403	648 (46%)	227 (16%)	717 (51%)	10.1	61	81.4	71.8
0.b	1 764	864 (49%)	244 (14%)	612 (35%)	10.7	60	82.1	73.3
Total	4 394	2 059 (47%)	657 (15%)	1774 (40%)	10.5	59	81.5	73.8

Abbreviations: $\bar{\text{Age Censor.}}$, mean age at censoring among censored participants; $\bar{\text{Age Dementia}}$, mean age at dementia onset among individuals diagnosed with dementia; $\bar{\text{Age Enrol.}}$, mean age at enrollment; $\bar{\text{Education}}$, mean number of years of education; Deceased, number (percentage) of deceased participants; Demented, number (percentage) of participants diagnosed with dementia; Male, number (percentage) of male participants; *N*, number of individuals in the sample.

Following Equation (2), we impute missing memory observations $y_{i}(t)$ for individual i at time t after their dropout time $t_{iF_{i}}$ and up to the first of their (i) observed event time, or (ii) final scheduled follow‐up time $T_{i}$ using the corresponding posterior draws from joint model (3) with $m_{i}(t)$ substituted by (4):

(4) $$\begin{array}{ll} m_{i}(t) & =f^{0.a}(t-\bar{t})+\left\{\underset{d\in \{0.b,2,3,4,5\}}{\sum }I(d_{i}=d)f^{d}(t-\bar{t})\right\}\\ & \;+x_{i}{\left(t\right)}^{⊤}\beta +b_{0i}+b_{1i}(t-\bar{t})+\Delta {\left(t-t_{iF_{i}}\right)}_{+} \end{array}$$

Here, we allow memory development, represented by a smooth function of age, to depend on the dropout pattern while the covariates have common parameters β across the patterns.

We also consider three assumptions about the missing memory data for sensitivity analyses. First, we assume that memory development post‐dropout adheres to the same function of age and missing data pattern as before dropout, without any additional post‐dropout memory decline, i.e., Δ=0. This corresponds to a form of missing not at random, since the memory trajectories depended on missing data patterns, and hence on the response indicator r.

**FIGURE B1:**
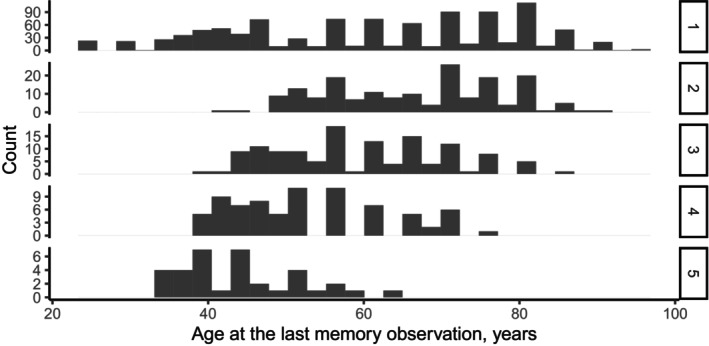
Histogram of ages at the last memory observation by the number of missing memory measurements for dropouts.

Secondly, we consider missing memory measures for subjects dropping out before the age of 60 years, to follow the same age trend as before dropout, i.e., Δ=0. Conversely, for those who dropped out after 60, their memory is assumed to decline by an additional 1 point per year (Δ=−1, note that the maximum of the memory score is 76) compared to the memory trajectory estimated from the measurements before dropout. The cut‐off of 60 years is chosen since missingness is thought to be more informative (memory is more strongly related to the dropout mechanism) at older ages. The cut‐off is also dictated by the Betula data: Figure B1 shows that it is mostly people younger than 60 years old at dropout had five missing memory measurements (only one memory observation available). If imputing with additional decline after dropout for these people, their memory decline would have been estimated as the most severe among all individuals, which is not plausible given the young age of these participants. Furthermore, these participants, who only engaged in the Betula study once, are more prone than older participants to drop out due to factors other than memory decline, such as time constraints related to participation.

Finally, we consider an accelerated additional memory decline after dropout, represented by allowing for different Δs for different ages. In our analyses, we use $\Delta =-{\left((\text{age}-25)/75\right)}^{3},$ where additional memory decline before the age of 60 years old is nearly zero and becomes increasingly bigger as a function of age. Here, for a hypothetical individual, the memory at age 25, the youngest age in Betula, is imputed using Δ=0, while the memory at 100 years old for this hypothetical individual is imputed using Δ=−1.

**FIGURE 2:**
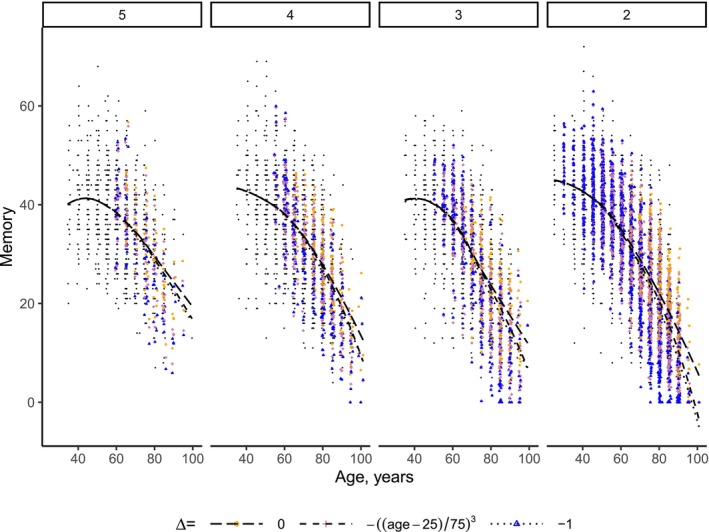
Observed (black) and posterior means over 3 000 posterior draws for missing memory scores for Δ=0 (orange), age‐varying $\Delta =-{\left((\text{age}-25)/75\right)}^{3}$ (pink), and Δ=−1 (blue) per missing data pattern d for patterns with imputed data (no data is imputed for patterns “0.a”, “0.b”). The curves represent LOESS smooth approximation of the mean memory.

Figure 2 illustrates how decreasing Δ results in a lower mean memory. The biggest differences between the approximations are for missing data pattern 2 with the highest proportion of data imputed. As per construction, the imputations before the age of 60 do not depend on Δ. Also, the predictions for patterns 0.a and 0.b do not depend on Δ because no data are imputed for them. Figure D1 shows that, as expected, the posterior means of memory score decreases with more negative Δ for those who dropped out after 60 years old.

In practice, we follow the Bayesian framework for imputation, as described below. We first fit joint model (3) with $m_{i}(t)$ substituted by (4) to the observed data using JMbayes2 R‐package and three chains (package's default), 50 000 warm‐up iterations, and 50 000 iterations afterward. We save every 50th MCMC iteration as posterior draws for fixed and random effects from each chain, resulting in a total of 3 000 posterior draws (1 000 draws for each chain). Note that for the observed data $\Delta {\left(t-t_{iF_{i}}\right)}_{+}$ is 0 since times t are less than dropout times $t_{iF_{i}}.$ For each scenario, corresponding to each specific assumption about Δ, we impute missing memory measurement K=5 times using (4) with the true values of model parameters substituted by five randomly selected posterior draws. The K=5 draws used for imputations are different for different values of Δ. If the suggested imputation memory score was below 0, we impute 0. We acknowledge the recommendations to use more imputations [29, 40]. However, we have chosen to use five imputations due to the long computational time (approximately 30 min for one scenario and one imputation). We accept a relative efficiency loss of around 4% for a 19% loss of information, given that 19% of longitudinal measurements are missing.

According to step C of the framework, after imputing missing longitudinal measurements, we fit the joint model (3) to each of K=5 completed data sets using JMbayes2 R‐package [39] with three chains (default setting), 20 000 warm‐up iterations, 30 000 iterations afterward, and use every 30th iterations after the warm‐up as posterior draws from each of five imputation iterations. For parameter pooling, we first combine the estimates from the three chains into one chain of size 3 000 for each imputation and combine the resulting chains using the parameter pooling approach for Bayesian analyses described in Appendix A.

### 4.4 Results

The estimates and the 95% credible intervals for the parameters of the joint model based on the observed data and the three considered assumptions about missing data are presented in Table 3. The corresponding standard errors are presented in Table D1. The posterior chains converged well as the scale reduction factor was estimated to be less than 1.11 for the fixed effects of the joint model and the variance of errors in the longitudinal submodel. The acceptance rates varied between 0.4 and 0.5 for all chains.

**TABLE 3:** Estimates (95% credible intervals) for the parameters of the joint model (3) fitted to the observed data and while accounting for missing longitudinal data according to multiple imputation procedure in Section 3 for different values of Δ.

	Estimate (CI)			
Predictor	Observed data	MI, Δ=0	MI, age‐varying Δ	MI, Δ=−1
Longitudinal submodel				
(Intercept)	31.93 (30.09, 33.86)	32.59 (30.9, 34.3)	35.62 (33.9, 37.34)	35.55 (33.81, 37.31)
ns($t-\bar{t}$, df = 3)1	−4.37 (−5.72, −3.11)	−6.03 (−7.32, −4.72)	−8.07 (−9.3, −6.85)	−8.42 (−9.62, −7.23)
ns($t-\bar{t}$, df = 3)2	−20.17 (−23.92, −16.56)	−23.62 (−26.87, −20.46)	−33.44 (−36.86, −30.08)	−35.86 (−39.24, −32.5)
ns($t-\bar{t}$, df = 3)3	−29.64 (−31.61, −27.62)	−33.14 (−35.07, −31.33)	−42.5 (−44.37, −40.7)	−47.15 (−48.93, −45.41)
Male	−2.9 (−3.36, −2.44)	−2.9 (−3.35, −2.45)	−2.93 (−3.38, −2.48)	−2.92 (−3.37, −2.46)
Education	0.83 (0.76, 0.89)	0.83 (0.77, 0.9)	0.84 (0.77, 0.9)	0.85 (0.78, 0.92)
First	−3.92 (−5.08, −2.64)	−4.19 (−5.31, −3.02)	−4.91 (−5.98, −3.84)	−5.43 (−6.43, −4.37)
First*age	0.04 (0.02, 0.06)	0.05 (0.03, 0.07)	0.06 (0.04, 0.08)	0.07 (0.05, 0.09)
Cohort	0.11 (0.09, 0.14)	0.09 (0.06, 0.11)	0.02 (0, 0.05)	0.01 (−0.02, 0.03)
Survival submodel				
Male	−0.48 (−0.77, −0.2)	−0.47 (−0.76, −0.18)	−0.42 (−0.71, −0.14)	−0.36 (−0.65, −0.09)
Education	0.04 (0.01, 0.08)	0.03 (0, 0.06)	0.02 (−0.01, 0.05)	0.01 (−0.02, 0.05)
Value(EM)	−0.07 (−0.09, −0.05)	−0.06 (−0.08, −0.04)	−0.05 (−0.07, −0.04)	−0.04 (−0.06, −0.02)
Slope(EM)	−2.57 (−3.49, −1.65)	−4.56 (−5.53, −3.58)	−3.2 (−4.08, −2.34)	−2.56 (−3.52, −1.66)

*Note:* Missing data patterns are ignored while fitting the substantive model to the observed data, while the fit of the substantive model under Δ=0 uses data imputed under the pattern‐mixture model, which considers missing data patterns. ns($t-\bar{t}$, df = 3)*i*: The *i*th vector in a natural cubic spline basis.

Abbreviations: Cohort, The difference between the participant's birth year and 1 937; Education, Years of education; EM, episodic memory score; First, The indicator of the first measurement; Male, 1 for males, 0 for females; t, Age; $\bar{t}$, The mean age at memory assessments with observed memory scores.

The results, based on 95% credible intervals, revealed that, on average, males were estimated to have poorer memory performance than females; higher baseline education corresponded to a better memory level. There were practice effects, i.e., participants were estimated to perform more poorly at the first measurement compared to the follow‐ups. The interaction of practice effects and age suggested that older people improved less from the first to the second measurement compared to younger people. The credible intervals for these relationships did not include zero regardless of the considered missing data mechanism. The regression parameter estimate for the birth cohort decreases with Δ, however, interpreting this decrease in isolation is challenging due to the strong correlation of approximately −0.86 between the birth cohort and age.

In the time‐to‐event model, females were estimated to have a higher hazard of dementia than males. The relationship between baseline education and the risk of dementia attenuated with more extreme changes after dropout where the 95% credible intervals included 0. The association between dementia risk and the memory level and speed of memory decline 5 years prior remained negative in the considered analyses. Worse memory levels and steeper memory decline were associated with a higher risk of dementia for all considered assumptions about the missing data. We also investigated the sensitivity of the results to cutoffs at 55 and 65 years instead of 60 years old and found that the relationship between dementia risk and memory was not materially affected by such cutoff choices (see Table D2).

**FIGURE 3:**
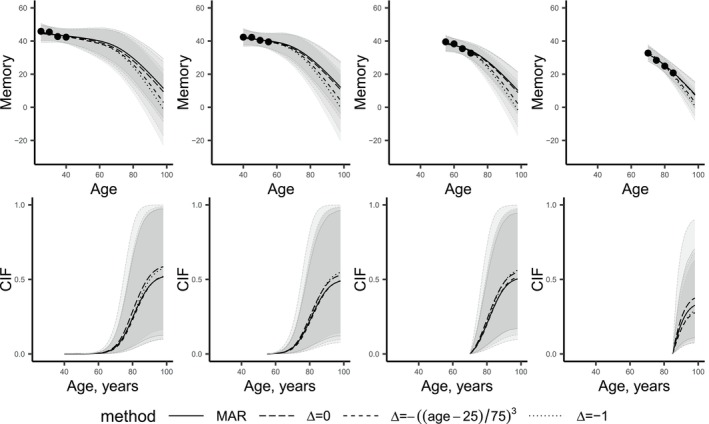
Observed data and dynamic prediction of memory (top row) and cumulative incidence function (CIF, bottom row) from the joint model fitted to the observed data (MAR) and while accounting for missing data for four new female individuals born in 1 937 with 10.5 years of education (mean education in the Betula data) that are dementia‐free at the last available memory measurement. The first woman has measurements only at 25, 30, 35, and 40 years of age; the second has measurements only at 40, 45, 50, and 55 years of age; the third woman has measurements only at 55, 60, 65, 70 years of age; the fourth woman has measurements at 70, 75, 80, 85 years of age. The measurements at ages 25,30,…,85 were equal to the mean of the observed memory scores at baseline and follow‐ups among the participants aged (22.5,27.5),(27.5,32.5),…,(82.5,87.5). Shaded areas represent credible intervals for the predicted memory and cumulative incidence function by combining 1 000 draws from each completed data set according to Appendix A.

**FIGURE D2:**
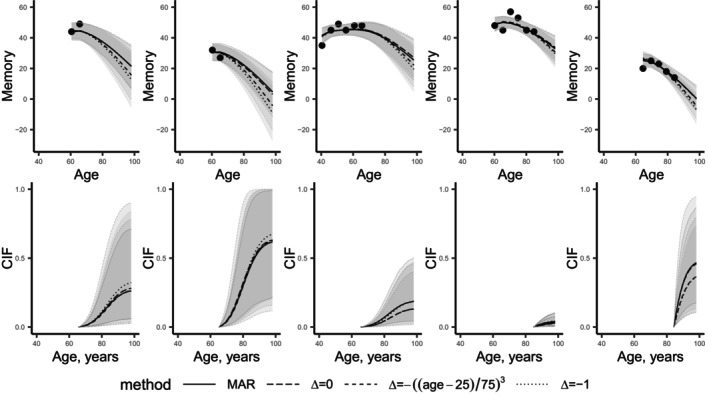
Observed data and dynamic prediction of memory (top row) and cumulative incidence function (CIF, bottom row) from the joint model fitted to the observed data (MAR) and while accounting for missing data for five new female individuals that are similar to some individuals in the Betula data born with 9–11 years of education that are dementia‐free at the last available memory measurement. Shaded areas represent credible intervals for the predicted memory and cumulative incidence function by combining 1 000 draws from each completed dataset according to Appendix A.

Figure 3 illustrates how different considered assumptions about the nature of missing data used to fit the models affect the dynamic predictions of memory function and survival for several new hypothetical “average” individuals, whose memory is equal to the corresponding mean memory in the Betula data and who were dementia‐free at their last memory measurement. To illustrate how predictions would look like for “real” patients, Figure D2 shows dynamic predictions for new individuals that have the same data as specific individuals in the Betula data. Note here that the observed longitudinal data for these new individuals stayed the same regardless of the model used to calculate the predictions. The difference between predictions is due to the substantive model being fit to different data. Under MAR, the substantive model was fitted to the observed Betula data alone (no imputation), while in the sensitivity analyses, the model was fitted to the completed after multiple imputation data sets. In each completed data set, only memory data changed while the survival data remained the same as in the observed data. The variation in survival predictions stemmed, among others, from differences in the estimated baseline hazards, survival model parameter estimates, as well as the estimates for the value and slope of memory changes. As expected, the predicted memory decline accelerated with more negative Δ since the fit is based on worse imputed memory. The prediction of the cumulative incidence of dementia was characterized by high uncertainty, as indicated by the wide credible intervals. The changes in the predicted incidence of dementia due to the considered sensitivity analyses were small compared to the uncertainty in the estimation of the hazards. As expected, worse observed memory was associated with a higher hazard of dementia (see individuals represented by the first, second, and the last three columns in Figure D2).

Our analysis found it challenging to determine the absolute value and direction of bias when predicting dementia risk for a new subject assuming MAR instead of MNAR data. For example, the predicted cumulative incidence of dementia from the scenario with the sharpest memory decline after dropout (Δ=−1) was the highest for some new subjects (see the second column in Figure 3), while for others this scenario resulted in lower risk (see the fourth column in Figure 3). Hence, our investigation did not reveal one factor that explains the relative position of the incidence predictions. In particular, further investigation showed the results were not driven by negative/positive random effects or higher/lower than average memory levels. The difference between the predictions may be related to the ages of a patient at which the longitudinal measurements are available. By construction, the imputations of the longitudinal data before 60 years old are not affected by sensitivity parameter Δ. For new people that have observed longitudinal data only at younger ages (columns 1 and 2 in Figure D2), the survival predictions at older ages are primarily informed by the imputed data at older ages, and as expected, a higher hazard of dementia is predicted from the models fitted to more sharply declining memory trajectories. Conversely, for new people with longitudinal data observed at older ages, their data are more optimistic compared to all other data used to fit the substantive models under Δ=−1 than under Δ=0. Therefore, the risk of dementia is estimated more optimistically when Δ=−1.

## 5 Discussion

This paper contributes to the application of disease prediction for new subjects from previously collected longitudinal health‐related data. A pressing problem arises when predictions are derived from models fitted to incomplete longitudinal datasets with MNAR missingness, which may severely distort models' predictive performance. In the present study, we proposed an accessible and practical sensitivity analysis approach to investigate the robustness of inferences from the joint models to different assumptions about missing data. We applied the approach to longitudinal data from the Betula study and found that level and change in episodic memory are informative for predicting dementia risk five years later. We further explored the robustness of results to various assumptions regarding the missingness mechanism and found the associations to be weaker for larger departures from MAR. Our sensitivity analyses showed that if considering missing not at random data, future predictive analyses might consider modifying the regression weights of memory when predicting dementia risk.

From a methodological perspective, this paper presents a multiple‐imputation‐based pattern‐mixture approach to perform sensitivity analyses in the joint modeling of longitudinal and time‐to‐event data. Pattern‐mixture modeling offers an intuitive method for capturing variations in the full‐data distributions by acknowledging that the distribution of longitudinal and time‐to‐event data may differ among various missing data patterns. By incorporating sensitivity parameters, the approach allows representing deviations from the standard missing at random (MAR) assumption. In this paper, the sensitivity parameter accounts for an additional decline in the longitudinal outcome after dropout (but note that Δ=0 corresponds to an MNAR assumption too, because the imputation varies between the missing data pattern). This interpretation facilitates a clearer understanding of the differences between the imputed and the observed values, which is essential for effective research communication. As with any method, the pattern‐mixture approach has some drawbacks. When data has many missing patterns, as in the Betula study, the pattern‐mixture approach requires the specification of many unidentified parameters. Moreover, the model can be unidentified as, for example, a mixed effect model with random intercept and slope for the pattern with only one observation per person. To reduce the number of parameters, one might need to define the relationships between the unidentified parameters or combine the missing data patterns, [25, 27] as this study did. Such adjustments introduce additional assumptions about data. Also, choices must be made about trajectories after dropout and intermittent missing through the choice of sensitivity parameters: Should they depend on the covariates, timing of dropout, be correlated, etc.? Additionally, pattern‐mixture models are designed to specify data models conditional on the missing data pattern, whereas the primary focus is often on the model's marginalized overall patterns.

An alternative to the proposed multiple imputation approach is a fully Bayesian analysis. The motivation for the multiple imputation approach lies in the separation of the imputation and analysis of the substantive model. The separation allows for the inclusion of additional information in the imputation model that is not in the substantive model. Also, an important practical advantage of this separation is that it enables analysts to use existing joint‐modeling software to fit the substantive and the imputation models, such as JMbayes2 R package instead of more challenging specification of full likelihood in the software for one‐step fully Bayesian inferences.

In the present study, we only considered missingness in the longitudinal outcome (episodic memory), and not missingness in the time‐to‐event outcome (dementia), since this was the case in the Betula data, where dementia diagnoses were obtained from medical records even after dropout. However, further research might consider other settings where dementia diagnosis after dropout is unobserved. In such cases, for example, dropout can be modeled as a competing risk [24, 41] or the time‐to‐event outcome can be also imputed. Additionally, a natural extension of the proposed approach is to consider a multi‐state illness‐death model, with “healthy”, “demented,” and “dead” states. Such a model would require modification of the substantive model.

The proposed approach can easily be modified for other research settings. For example, we chose to infer a lag‐5 dependency between the true memory level, the speed of memory decline, and time‐to‐dementia. Alternative association structures and lags can also be considered. Moreover, in the sensitivity analyses, we solely considered changes in the linear slope after dropout despite non‐linear memory trajectories. Non‐linear and more individualized adjustments post‐dropout could also be explored, for example, changes depending on the missing data pattern (which would require introducing multiple sensitivity parameters into the model), relative changes, etc., achieved by appropriately modifying the imputation model.

## Author Contributions

The work was led by Tetiana Gorbach who performed all statistical analysis. All authors contributed to the conceptualization of the research, interpretation of the results, and writing the manuscript.

## Disclosure

The authors have nothing to report.

## Conflicts of Interest

The authors declare no conflicts of interest.

## Supporting information


**Supplementary Information.** Supplementary Information.

## Data Availability

Data from the Betula project cannot be made publicly available due to ethical and legal restrictions. However, access to data is available after approval by the Steering Group of the Betula project (https://www.umu.se/en/betula). Access to data by qualified investigators is subject to scientific and ethical review and must comply with the European Union General Data Protection Regulations (GDPR)/all relevant guidelines. The completion of a data transfer agreement (DTA) signed by an institutional official will be required. Please see more instructions at https://www.umu.se/en/research/projects/betula‐aging‐memory‐and‐dementia/collaboration‐on‐betula‐data/.

## Appendix A Parameter Pooling

In step 3 in Section 3, estimates of a quantity of interest and their variances, ${\left\{{\hat{Q}}_{k},U_{k}\right\}}_{k=1}^{K}$ from fitting the substantive model to the K completed data sets are combined for final inference. The quantity of interest can be a substantive model parameter, for example, the association α between the longitudinal measures and the hazards of dementia in the model (1), or some other known function of the data.

In the frequentist approach, the scalar parameter estimates that are normally distributed can be combined using Rubin's rules [31]. The combined estimate is $\bar{Q}=\frac{1}{K}\sum_{k=1}^{K}{\hat{Q}}_{k}.$ The estimate of the total variance T of the combined estimate $\bar{Q}$ is the weighted sum of variances within each of K completed data sets and the variance between the completed data sets: $T=Ū+\left(1+\frac{1}{K}\right)B.$ Here, the within‐variance is $Ū=\frac{1}{K}\sum_{k=1}^{K}{Ū}_{k},$ and the between‐variance is $B=\frac{1}{K-1}\sum_{k=1}^{K}{\left({\hat{Q}}_{k}-\bar{Q}\right)}^{2}.$ The 100(1−α)% confidence interval of $\bar{Q}$ can be calculated as $\bar{Q}\pm t_{\nu ,1-\alpha /2}\sqrt{T},$ where $t_{\nu ,1-\alpha /2}$ is the quantile corresponding to probability 1−α/2 of the t‐distribution with $\nu =\frac{K-1}{{\left(\frac{B+B/K}{T}\right)}^{2}}$ degrees of freedom (Equation 3.1.6 in Rubin [31]). Non‐normal parameters can be transformed toward normality before applying Rubin's rules and then back‐transformed (see Section 5.2 in van Buuren [32]).

Using a Bayesian approach, respective pooled summaries for quantity Q can be constructed by firstly sampling J values of Q from the posterior $f(Q|D^{k})$ for each of the completed data set $D^{k}$, k=1,…,K and where J is some large number [34]. We denote these J draws as $\hat{f}(Q^{k}).$ Secondly, the draws $\hat{f}(Q^{k}),k=1,\ldots ,K$ are combined to obtain a set of all posterior draws $\hat{f}(Q^{all})$ of size JK. The point estimate of Q can be the mean or median of posterior draws $\hat{f}(Q^{all}).$ The (1−α)100% credible intervals can be defined as $(p_{\alpha /2},p_{1-\alpha /2}),$ where $p_{\alpha /2}$ and $p_{1-\alpha /2}$ are the $2.5^{th}$ and $97.5^{th}$ percentiles of $\hat{f}(Q^{all})$.

## Appendix B Data Preparation

• Each row in a data file represents an observation of memory and health for a specific participant for a specific Betula wave.
• From a data file, delete 1 374 rows with no data for any of the five memory tests (9 733 rows for 4 424 people left).
• Define
  · sex to be 0 for females and 1 for males.
  · memory score as the sum of scores for five episodic memory tasks: Immediate free recall of 16 short sentences without enactment, immediate free recall of 16 short sentences with enactment, delayed cued recall of nouns from the previously presented sentences without enactment, delayed cued recall of nouns from the enacted sentences, immediate free recall of a list of 12 orally presented nouns. The memory scores range from 0 to 76.
  · age at memory assessment for each Betula wave as rounded to one decimal age at memory test if available; for 85 rows with no information on the memory test, define age as rounded to one decimal age at health test.
  · date of testing as the date of memory test (using year, month, and the first day of the month if the testing was performed in the first half of the month, otherwise the 15th of the month) if available; otherwise, the date of health test, defined similarly using year, month and a half of the testing month.
  · date of birth by subtracting age from date of testing.
  · age at death as a sum of age at testing and the difference between the date of death and the age at testing.
  · age at death evaluation as the sum of age and the difference between the date when the participants' vital status was last checked and the date of testing.
  · age at dementia evaluation as a sum of age and the difference between the date of the latest dementia diagnostic evaluation and the date of testing.
  · time to impute the memory measures as the time at the last follow‐up plus 5 years multiplied by the order of missing measure (1 for the first missing measure, 2 for the second, etc.)
• Delete data for 11 subjects (20 rows, 9 713 rows for 4 413 people left) with early onset of dementia. Individuals with early onset dementia are excluded since they have a more aggressive disease progression, and the long time span of 5 years between the Betula waves does not allow capturing this progression.
• Delete 197 rows with missing scores for some of the five tests used to calculate the memory score (9 516 rows for 4 394 people are left).
• Define age at the event as age at dementia onset if available (657 out of 4 394 people), otherwise age at censoring. Age at censoring is defined as the minimum age of death and age at the latest dementia evaluation, if available (3663 people); for 74 participants with no date of death or latest dementia evaluation, the age at censoring is defined as the age at memory assessment plus 0.003 years (equals approximately a day).
• Use baseline education instead of time‐dependent education. If baseline education is unavailable, we have checked if any information about education is available at other assessment waves. One participant had two observations of 5 years of education; we used five as their years of education. For three participants, zero years of education was recorded at the sixth Betula wave (this could be the data or denote no info about education if measurement or processing error; the reason for zero is not stated in the variable explanation file, so we use 0).
• For one subject, substitute their highly unlikely value of education of 70 years to not available.
• Delete 94 longitudinal observations after dementia onset (9 422 rows for 4 394 participants are left).
• Delete 83 observations (63 participants, 9 339 rows in the longitudinal data, and 4 331 rows in the survival data left) with missing education since education is one of the covariates in the model.

**TABLE B1:** Number of participants per missing data pattern and Betula waves with observed memory and the waves with observed memory before dementia onset.

Missing data pattern d	Betula cohort	Observed waves	Observed waves before dementia	Number of participants
0.a	S1	123456	12345	4
0.a	S1	123456	123456	209
0.a	S1	12346	12346*	3
0.a	S1	12356	12356*	4
0.a	S1	12456	12456*	1
0.a	S1	1256	1256*	1
0.a	S3	23456	23456	220
0.a	S3	2346	2346*	6
0.a	S3	2356	2356*	2
0.a	S3	2456	2456*	1
5	S1	12345	1234	6
5	S1	12345	12345	127
5	S1	1235	1235*	3
5	S1	1245	1245*	4
5	S1	125	125*	2
4	S1	1234	12	1
4	S1	1234	123	10
4	S1	1234	1234	127
4	S1	124	12	2
4	S1	124	124*	4
4	S1	134	134*	1
4	S3	2345	234	1
4	S3	2345	2345	148
4	S3	235	235*	9
4	S3	245	2	1
4	S3	25	25*	2
4	S3	345	34	1
4	S3	345	345	1
3	S1	123	12	10
3	S1	123	123	121
3	S1	13	13*	5
3	S3	234	23	14
3	S3	234	234	162
3	S3	24	2	2
3	S3	24	24*	11
3	S3	34	34	1
2	S1	1	1	156
2	S1	12	1	13
2	S1	12	12	185
2	S2	2	2	383
2	S3	2	2	208
2	S3	23	2	15
2	S3	23	23	148
2	S3	3	3	2
2	S6	5	5	293
0.b	S2	23	2	11
0.b	S2	23	23	573
0.b	S2	235	23	1
0.b	S2	235	235	6
0.b	S2	25	25	1
0.b	S2	3	3	5
0.b	S4	3	3	546
0.b	S4	35	35	1
0.b	S5	4	4	557
0.b	S6	56	56	63
Total				4394

*Note:* Missing memory measurements are represented by missing corresponding Betula wave numbers in “Observed waves” and “Observed waves before dementia”.

* Denotes the patterns with intermediate missing (S2 patterns 23 and 235 as well as S4 pattern 35 do not have an intermediate missing since S2 and S4 were scheduled only for one measurement but some people participated more than once).

**TABLE B2:** Number of participants per Betula cohort and missing data pattern.

Betula cohort	Missing data pattern d	Number of participants
S1	0.a	222
S1	5	142
S1	4	145
S1	3	136
S1	2	354
S2	2	383
S2	0.b	597
S3	0.a	229
S3	4	163
S3	3	190
S3	2	373
S4	0.b	547
S5	0.b	557
S6	2	293
S6	0.b	63
Total		4 394

## Appendix C Cognitive Trajectory Modeling

To select the model for longitudinal memory trajectories, we compared the fit of several linear mixed effects models to the observed data. In the models, lme_fit_1—lme_fit_5, memory y(t) at age t depended on the polynomial of the age of the first, second, third, fourth, and fifth degree. We also compared these parametric models to less restricted models lme_fit_ns2—lme_fit_ns5, where the age‐memory relationship was modeled through a smooth function via a natural cubic spline with 1, 2, 3, 4, and 5 internal knots (2–6 degrees of freedom, respectively). In all compared models, we included the following covariates: Sex, baseline education, age cohort defined as the difference between the participant's birth year and 1 937, an indicator of enrollment to estimate practice effects and interaction of age, and the enrollment indicator to represent the dependency of practice effects on age at the first measurement. Each model included a random intercept and a random linear slope for each participant.

The compared models are

(C1)
$$\begin{array}{ll} \text{lme\_fit\_1:}\;y_{i}(t) & =\beta_{0}+x_{i}{\left(t\right)}^{⊤}\beta_{1}+\beta_{2}(t-\bar{t})\\ & \;+b_{0i}+b_{1i}(t-\bar{t})+\varepsilon_{i}(t)\\ \text{lme\_fit\_2:}\;y_{i}(t) & =\beta_{0}+x_{i}{\left(t\right)}^{⊤}\beta_{1}+\beta_{2}(t-\bar{t})\\ & \;+\beta_{3}{\left(t-\bar{t}\right)}^{2}+b_{0i}+b_{1i}(t-\bar{t})+\varepsilon_{i}(t)\\ \text{lme\_fit\_3:}\;y_{i}(t) & =\beta_{0}+x_{i}{\left(t\right)}^{⊤}\beta_{1}+\beta_{2}(t-\bar{t})+\beta_{3}{\left(t-\bar{t}\right)}^{2}\\ & \;+\beta_{4}{\left(t-\bar{t}\right)}^{3}+b_{0i}+b_{1i}(t-\bar{t})+\varepsilon_{i}(t)\\ \text{lme\_fit\_4:}\;y_{i}(t) & =\beta_{0}+x_{i}{\left(t\right)}^{⊤}\beta_{1}+\beta_{2}(t-\bar{t})+\beta_{3}{\left(t-\bar{t}\right)}^{2}\\ & \;+\beta_{4}{\left(t-\bar{t}\right)}^{3}+\beta_{5}{\left(t-\bar{t}\right)}^{4}+b_{0i}\\ & \;+b_{1i}(t-\bar{t})+\varepsilon (t)\\ \text{lme\_fit\_5:}\;y_{i}(t) & =\beta_{0}+x_{i}{\left(t\right)}^{⊤}\beta_{1}+\beta_{2}(t-\bar{t})\\ & \;+\beta_{3}{\left(t-\bar{t}\right)}^{2}+\beta_{4}{\left(t-\bar{t}\right)}^{3}+\beta_{5}{\left(t-\bar{t}\right)}^{4}\\ & \;+\beta_{6}{\left(t-\bar{t}\right)}^{5}+b_{0i}+b_{1i}(t-\bar{t})+\varepsilon_{i}(t)\\ \text{lme\_fit\_ns2:}\;y_{i}(t) & =f(t-\bar{t},df=2)+x_{i}{\left(t\right)}^{⊤}\beta +b_{0i}\\ & \;+b_{1i}(t-\bar{t})+\varepsilon_{i}(t)\\ \text{lme\_fit\_ns3:}\;y_{i}(t) & =f(t-\bar{t},df=3)+x_{i}{\left(t\right)}^{⊤}\beta +b_{0i}\\ & \;+b_{1i}(t-\bar{t})+\varepsilon_{i}(t)\\ \text{lme\_fit\_ns4:}\;y_{i}(t) & =f(t-\bar{t},df=4)+x_{i}{\left(t\right)}^{⊤}\beta \\ & \;+b_{0i}+b_{1i}(t-\bar{t})+\varepsilon_{i}(t)\\ \text{lme\_fit\_ns5:}\;y_{i}(t) & =f(t-\bar{t},df=5)+x_{i}{\left(t\right)}^{⊤}\beta \\ & \;+b_{0i}+b_{1i}(t-\bar{t})+\varepsilon_{i}(t)\\ \text{lme\_fit\_ns6:}\;y_{i}(t) & =f(t-\bar{t},df=6)+x_{i}{\left(t\right)}^{⊤}\beta \\ & \;+b_{0i}+b_{1i}(t-\bar{t})+\varepsilon_{i}(t) \end{array}$$

Random effects $b_{i}=(b_{0i},b_{1i})$ were assumed to be normally distributed with variance‐covariance matrix D independent from the error terms $\varepsilon_{i}.$ Errors $\varepsilon_{i}(t)$ were assumed to be uncorrelated. All models were fitted using lme function within nlme R‐package [42].

Table C1 provides measures of the fit of the considered models to the observed data and their comparisons. The model with a smooth function of age three degrees of freedom (two internal knots) lme_fit_ns3 provided the best fit to the data according to AIC and BIC.

**TABLE C1:** Longitudinal model comparison.

	Model	df	AIC	BIC	logLik	Test	L.Ratio	p‐value
lme_fit_1	1	11.00	62712.57	62791.13	−31345.28			
lme_fit_2	2	12.00	62287.12	62372.82	−31131.56	1 versus 2	427.45	0.00
lme_fit_3	3	13.00	62240.97	62333.81	−31107.48	2 versus 3	48.15	0.00
lme_fit_4	4	14.00	62240.08	62340.07	−31106.04	3 versus 4	2.88	0.09
lme_fit_5	5	15.00	62240.02	62347.15	−31105.01	4 versus 5	2.07	0.15
lme_fit_ns2	6	12.00	62281.67	62367.38	−31128.84	5 versus 6		
lme_fit_ns3	7	13.00	62233.84	62326.69	−31103.92	6 versus 7		
lme_fit_ns4	8	14.00	62235.39	62335.38	−31103.70	7 versus 8		
lme_fit_ns5	9	15.00	62236.43	62343.56	−31103.22	8 versus 9		
lme_fit_ns6	10	16.00	62237.67	62351.94	−31102.84	9 versus 10		

Abbreviations: AIC, Akaike information criterion; BIC, Bayesian information criterion; df, Degrees of freedom in the model; L. Ratio: Likelihood ratio for the likelihood‐ratio test performed; logLik, Log‐likelihood, Test indicates what models were compared in a corresponding likelihood‐ratio test.

## Appendix D Some Illustrations of Imputations and Inferences for the Substantive Model

**TABLE D1:** Standard errors of the estimates for joint model (3) fitted to the observed data and while accounting for missing longitudinal data according to multiple imputation procedure in Section 3 for different values of Δ.

	Standard errors			
Predictor	Observed data	MI, Δ=0	MI, age‐varying Δ	MI, Δ=−1
Longitudinal submodel				
(Intercept)	0.94	0.88	0.88	0.89
ns($t-\bar{t}$, df = 3)1	0.66	0.67	0.63	0.61
ns($t-\bar{t}$, df = 3)2	1.88	1.65	1.72	1.71
ns($t-\bar{t}$, df = 3)3	1.04	0.96	0.93	0.90
Male	0.23	0.23	0.23	0.23
Education	0.03	0.03	0.03	0.03
First	0.63	0.59	0.55	0.53
First * age	0.01	0.01	0.01	0.01
Cohort	0.01	0.01	0.01	0.01
Survival submodel				
Male	0.15	0.15	0.15	0.14
Education	0.02	0.02	0.02	0.02
Value(EM)	0.01	0.01	0.01	0.01
Slope(EM)	0.46	0.50	0.44	0.48

*Note:* Missing data patterns are ignored while fitting the substantive model to the observed data, while the fit of the substantive model under Δ=0 uses data imputed under the pattern‐mixture model, which considers missing data patterns.

Abbreviations: Cohort, The difference between the participant's birth year and 1 937; Education, Years of education; First, The indicator of the first measurement; Male, 1 for males, 0 for females; ns($t-\bar{t}$, df = 3)*i*, The *i*th vector in a natural cubic spline basis; *t*, Age; $\bar{t}$, The mean age at memory assessments with observed memory scores.

**TABLE D2:** Estimates (95% credible intervals) for the parameters of the joint model (3) fitted to data while accounting for missing longitudinal data according to multiple imputation procedure in Section 3 with Δ=−1 for those who drop out after 55 years, 60 years, and 65 years old.

	Estimate (CI)		
	MI, Δ=−1		
Predictor	Cut‐off 55	Cut‐off 60	Cut‐off 65
Longitudinal submodel			
(Intercept)	36.49 (34.57, 38.57)	35.55 (33.81, 37.31)	35.47 (33.63, 37.34)
ns($t-\bar{t}$, df = 3)1	−10.58 (−12.05, −9.19)	−8.42 (−9.62, −7.23)	−7.41 (−8.67, −6.17)
ns($t-\bar{t}$, df = 3)2	−37.59 (−41.44, −33.92)	−35.86 (−39.24, −32.5)	−35.37 (−39.12, −31.68)
ns($t-\bar{t}$, df = 3)3	−48.44 (−50.36, −46.62)	−47.15 (−48.93, −45.41)	−45.75 (−47.77, −43.77)
Male	−2.92 (−3.38, −2.46)	−2.92 (−3.37, −2.46)	−2.91 (−3.37, −2.47)
Education	0.85 (0.78, 0.92)	0.85 (0.78, 0.92)	0.85 (0.78, 0.91)
First	−5.56 (−6.64, −4.46)	−5.43 (−6.43, −4.37)	−5.47 (−6.53, −4.43)
First*age	0.07 (0.05, 0.09)	0.07 (0.05, 0.09)	0.07 (0.05, 0.09)
Cohort	−0.03 (−0.06, 0)	0.01 (−0.02, 0.03)	0.03 (0, 0.05)
Male	−0.34 (−0.62, −0.06)	−0.36 (−0.65, −0.09)	−0.38 (−0.66, −0.1)
Education	0.01 (−0.03, 0.04)	0.01 (−0.02, 0.05)	0.02 (−0.02, 0.05)
Value(EM)	−0.03 (−0.05, −0.01)	−0.04 (−0.06, −0.02)	−0.05 (−0.06, −0.03)
Slope(EM)	−2.42 (−3.25, −1.6)	−2.56 (−3.52, −1.66)	−2.82 (−3.81, −1.9)
